# A comparison of residual diagnosis tools for diagnosing regression models for count data

**DOI:** 10.1186/s12874-020-01055-2

**Published:** 2020-07-01

**Authors:** Cindy Feng, Longhai Li, Alireza Sadeghpour

**Affiliations:** 1 School of Epidemiology and Public Health, Faculty of Medicine, University of Ottawa, 600 Peter Morand, Ottawa, K1G5Z3 Canada; 2 School of Public Health, University of Saskatchewan, 104 Clinic Place, Saskatoon, S7N2Z4 Canada; 3 Department of Mathematics and Statistics, University of Saskatchewan, 106 Wiggins Road, Saskatoon, S7N5E6 Canada

**Keywords:** Randomized quantile residual, Model checking, Model diagnostics, Goodness-of-fit test

## Abstract

**Background:**

Examining residuals is a crucial step in statistical analysis to identify the discrepancies between models and data, and assess the overall model goodness-of-fit. In diagnosing normal linear regression models, both Pearson and deviance residuals are often used, which are equivalently and approximately standard normally distributed when the model fits the data adequately. However, when the response vari*able is discrete, these residuals are distributed far from normality and have nearly parallel curves according to the distinct discrete response values, imposing great challenges for visual inspection.

**Methods:**

Randomized quantile residuals (RQRs) were proposed in the literature by Dunn and Smyth (1996) to circumvent the problems in traditional residuals. However, this approach has not gained popularity partly due to the lack of investigation of its performance for count regression including zero-inflated models through simulation studies. Therefore, we assessed the normality of the RQRs and compared their performance with traditional residuals for diagnosing count regression models through a series of simulation studies. A real data analysis in health care utilization study for modeling the number of repeated emergency department visits was also presented.

**Results:**

Our results of the simulation studies demonstrated that RQRs have low type I error and great statistical power in comparisons to other residuals for detecting many forms of model misspecification for count regression models (non-linearity in covariate effect, over-dispersion, and zero inflation). Our real data analysis also showed that RQRs are effective in detecting misspecified distributional assumptions for count regression models.

**Conclusions:**

Our results for evaluating RQRs in comparison with traditional residuals provide further evidence on its advantages for diagnosing count regression models.

## Background

Count data consist of non-negative integers that represent the number of times a discrete event is observed; for example, number of clinic visits, hospital admissions, adverse drug events, substance abuse and rates of cardiac arrest. Poisson regressions or negative binomial (NB) regressions are often used to model such data; however, this type of data may contain a large number of zero values that a Poisson or NB models can not adequately model. To overcome the issue with excessive zeros, zero inflated models [1] were proposed in literature to model the excessive number of zeros, which are a mixture of two components: a point mass at zero and a count regression model, such as a Poisson or NB model. Zero inflated models were widely used in many population and epidemiological studies [2–7]. Despite the advance in developing counts regression models, model diagnosis remains understudied and lacks of clarity on practical issues.

Examining residuals is a primary approach for identifying the overall discrepancies between models and data (e.g., non-linear effects, over-dispersion, zero-inflation), and observations that are not accommodated by the models (e.g., outliers). Residual analyses can also diagnose the overall goodness-of-fit (GOF) and adequacies of a model. Pearson and deviance residuals have been often used for diagnosing generalized linear models (GLMs) [8, 9]. Pearson residuals are defined as the standardized distances between the observed and expected responses, and deviance residuals are defined as the signed square root of the individual contributions to the model deviance (i.e., the difference between the log-likelihoods of the saturated and fitted models). In normal regressions, Pearson and deviance residuals are the same and asymptotically follow a normal distribution under the true model [8, 9]. In order to assess the model fit, these residuals are commonly plotted against the fitted values and each covariate, as well as compared against the standard normal distribution. The chi-squared (*χ*^2^) test statistic is often used to measure the overall GOF of a normal regression model. The *χ*^2^ statistic was proven to have an asymptotic *χ*^2^ distribution with *n*−*p* degrees of freedom $\left (\text {denoted by} \chi ^{2}_{n-p}\right)$ [8, 9]. However, in non-normal regression, specifically in data with the response variable being distributed on a small number of distinct values, the Pearson and deviance residuals do not typically follow (marginally and conditionally) a normal distribution. The plots of their residuals contain nearly parallel curves, making their assessment difficult to interpret due to the lack of a unified reference distribution for comparison. Furthermore, although the *χ*^2^ tests with $\chi ^{2}_{n-p}$ as the null distribution are widely used for quantitatively measuring the overall GOF in non-normal regression, the $\chi ^{2}_{n-p}$ distribution is often very poor for approximating the true null sampling distribution of *χ*^2^ statistic [8, 9] except for situations where the response variable is approximately normally distributed (e.g., Poisson with large means).

In a short communication paper, Dunn and Smyth [10] introduced the randomized quantile residual (RQR) method. The key idea of the RQRs is to introduce randomizations between the discontinuity gap of the cumulative distribution function (CDF) and then invert the fitted distribution function for each response value and finding the equivalent standard normal quantile. Dunn and Smyth [10] showed that the RQRs are approximately normally distributed under a correctly specified model. Klar and Meintanis (2012) [11] proposed the standardized RQRs for performing goodness-of-fit tests in generalized linear models, which was shown to be approximately standard normally distributed. For modeling non-normal and continuous outcome data, several studies have investigated the properties of quantile residuals for checking the model fit. For example, Pereira (2019) [12] investigated the properties of the quantile residual in the beta regression model and demonstrated that the distribution of the quantile residual is better approximated by the standard normal distribution than that of the other residuals in most scenarios. Lemonte and Moreno-Arenas (2019) [13] proposed the normalized quantile residual to check the adequacy of the generalized Johnson *S*_*B*_ (GJS) regression model, which were shown as a better choice to identify departures from the model assumptions and to assess the overall goodness-of-fit than the deviance residual. Scudilio and Pereira (2020) [14] proposed an adjusted quantile residual for diagnosing inverse Gaussian or Gamma regression models, which was shown to be a better choice to perform diagnostic analysis compared to traditional residuals. Despite the increasing awareness of RQRs, the literature on using RQRs has primarily focused on diagnosing generalized linear models with the distribution of the response variable belonging to the exponential family, i.e., Poisson, Negative binomial, inverse Gaussian, Gamma, or being continuous non-normal. A broader simulation study that evaluates the performance of RQRs for diagnosing count regression models, including zero-inflated count regressions would be useful for applied statisticians to understand the advantages of RQRs.

The purposes of this article are therefore to conduct simulation studies to (1) demonstrate that the RQRs approximately follow a normal distribution for count regression models when the model is correctly specified apart from the sampling variability in the estimated parameters, and (2) examine the power of the Shapiro-Wilk normality test for examining the performance of RQRs as an overall model diagnosis tool. More specifically, we show that the RQR method, in general, has high power and low type I error for detecting various model misspecification (i.e., having great statistical power in detecting many types of model inadequacy, such as non-linearity, zero-inflation, and over-dispersion).

For the remaining of this article, in “Methods” section, we review the commonly used count regression models and discuss the inadequacies of the Pearson and deviance residuals for detecting model misspecification followed by a brief review of RQRs. In “Results” section, the results of the simulation studies and a real data application are presented. More specifically, in “Simulation studies” section, we present the results of simulation studies to demonstrate the superior performance of RQRs for detecting various forms of model misspecification. In “Real data application” section, a real data application based on a health care utilization study is presented to illustrate the performance of RQRs in comparison with other traditional residuals. Implications and limitations of the study are presented in “Discussion” section. Concluding remarks are given in “Conclusion” sections.

## Methods

### Regression models for count data

#### Poisson and negative binomial (NB) models

Let *y*_*i*_ denote a discrete random variable following a Poisson distribution with mean *λ*_*i*_, *i*=1,⋯,*n*. The probability mass function (PMF) is given by $dpois(y_{i};\lambda _{i})=\frac {e^{-\lambda _{i}} \lambda _{i}^{y_{i}}}{y_{i} !}$, *y*_*i*_=0,1,2⋯ and the cumulative probability function (CDF) is given by $ppois(y_{i};\lambda _{i})=\sum _{y_{i}=0}^{y_{i}} dpois(y_{i};\lambda _{i})$. For a NB regression model with mean *λ*_*i*_ and shape parameter *k*, the PMF can be expressed as, $ dnb(y_{i};\lambda _{i}, k) = \frac {\Gamma (y_{i}+k)}{\Gamma (k) \Gamma (y_{i}+1)} \left (\frac {\lambda _{i}}{\lambda _{i}+k}\right)^{y_{i}} \left (\frac {k}{\lambda _{i}+k}\right)^{k}, y_{i}=0, 1, 2\cdots $ and the CDF can be expressed as $pnb(y_{i};\lambda _{i}, k) = \sum _{y_{i}=0}^{y_{i}} dnb(y_{i};\lambda _{i}, k). $ Poisson or NB models with a log link function can be then written as $ \text {log}(\lambda _{i}) = \boldsymbol {X}^{T}_{i} \boldsymbol {\beta }$, where ***X***_*i*_ denotes the design matrix of a set of covariates and ***β*** represents the corresponding regression coefficients.

#### Zero-inflated models

In practice, count data often contains excessive zeros that may not be accurately captured by a conventional Poisson or NB model; these data are commonly known as *zero-inflated* data. One popular approach to model such data is a mixture model of degenerate zeros from the non-risk group (i.e., structural zeros) and responses with random zeros or positive values from the at-risk group [1, 2, 5, 15–17]. For example, one might be interested in the question: “How often do you revisit hospitals within the last 30 days after being released from the hospital?” Among the patients reported zero number of hospital revisits, some may be fully recovered from this disease leading to genuine non-users (structural zeros); however, some might currently behave as non-users (sampling zeros) and would have the potential to revisit the hospital depending on their health status.

The zero-inflated Poisson (ZIP) model with parameters *λ*_*i*_ and *p*_*i*_, denoted by ZIP(*λ*_*i*_,*p*_*i*_), is defined by

1 $$ y_{i} \sim \left\{\begin{array}{ll} 0 & \quad \text{with probability} \ p_{i} \\ \text{Poisson}(\lambda_{i}) & \quad \text{with probability}\ 1-p_{i}, \end{array}\right.  $$

where *λ*_*i*_ is the mean of the Poisson component and *p*_*i*_ is the probability of belonging to the structural zeros component for the *i*th observation.

Let *dzip*(*y*_*i*_;*λ*_*i*_,*p*_*i*_) denote the PMF of the ZIP distribution with the unconditional probability distribution written as

2 $$\begin{array}{*{20}l} dzip(y_{i}=0) &= p_{i} +\left(1-p_{i}\right)e^{-\lambda_{i}} \end{array} $$

3 $$\begin{array}{*{20}l} dzip(y_{i}=j) &= \left(1-p_{i}\right)\frac{e^{-\lambda_{i}}\lambda_{i}^{j}}{j!}, \, \, j=1, 2, \ldots. \end{array} $$

The CDF, denoted by *pzip*(*y*_*i*_;*λ*_*i*_,*p*_*i*_), is then derived as

4 $$ \begin{aligned}&pzip\left(y_{i}=J;\lambda_{i},p_{i}\right)=\sum_{j=0}^{J} dzip(y_{i}=j)=p_{i}\\&\quad+(1-p_{i})ppois(J, \lambda_{i}), \end{aligned}  $$

where *ppois*(*J*,*λ*_*i*_) denotes the CDF of a Poisson distribution. The marginal mean and variance of a ZIP distribution can be derived as

5 $$ E(y_{i})= \mu_{i} = (1-p_{i})\lambda_{i}, \, \, \, V(y_{i})= \left(1-p_{i}\right)\lambda_{i} \left(1+p_{i} \lambda_{i} \right).  $$

As a result, zero-inflated model can accommodate overdispersion relative to a Poisson model, since *V*(*y*_*i*_)>*E*(*y*_*i*_).

The ZIP model can include covariates for modeling both *p*_*i*_ and *λ*_*i*_. Generally, *p*_*i*_ is modeled with a logistic regression and *λ*_*i*_ is modeled as a log-linear regression. ZIP model can be then written as,

6 $$\begin{array}{*{20}l} \text{logit}(p_{i}) =\boldsymbol{Z}^{T}_{i} \boldsymbol{\gamma} \, \, \text{and} \, \, \log (\lambda_{i}) = \boldsymbol{X}^{T}_{i} \boldsymbol{\beta}, \end{array} $$

where $\boldsymbol {Z}^{T}_{i}$ and $\boldsymbol {X}^{T}_{i}$ are vectors of covariates with corresponding vectors of regression parameters, ***γ*** and ***β***, for *p*_*i*_ and *λ*_*i*_, respectively. The zero-inflated negative binomial (ZINB) model can be defined similarly by replacing Poisson distribution with NB distribution in equation (1).

### Pearson residuals

The *Pearson residual*, defined as the raw residual scaled by the estimated standard deviation of the response variable, is the most common measure for GOF, which can be expressed as,

7 $$ r^{P}_{i} = \frac{y_{i}-\hat{\mu}_i}{\sqrt{\widehat{V}(y_{i})}},  $$

where $\hat {\mu }_{i}$ is the fitted value for *y*_*i*_ and $\widehat {V}(y_{i})$ is the estimation of variance for *y*_*i*_. Table 1 presents the Pearson residuals for some commonly used count regression models.

**Table 1:** Pearson residuals for commonly used regression models for count data

Model	Pearson residuals
Poisson	$r^{P}_{i} = \frac {y_{i}-\hat {\lambda }_{i}}{\sqrt {\hat {\lambda }_{i}}}$
NB	$r^{P}_{i} = \frac {y_{i}-\hat {\lambda }_{i}}{\sqrt {\hat {\lambda }_{i}+\hat {\lambda }_{i}^{2}/k}}$
ZIP	$r^{P}_{i} = \frac {y_{i}-\left (1-\hat {p}_i \right)\hat {\lambda }_i}{\sqrt {\left (1-\hat {p}_i \right)\hat {\lambda }_i \left [ 1+\hat {p}_i \hat {\lambda }_i \right ]}}$
ZINB	$r^{P}_{i} = \frac {y_{i}-(1-\hat p_{i})\hat \lambda _{i}}{\sqrt {(1-\hat p_{i})\left (\hat \lambda _{i}+\frac {{\hat \lambda _{i}}^{2}}{k}\right)+{\hat \lambda _{i}}^{2} \left ({\hat p_{i}}^{2}+\hat p_{i}\right)}}$

### Deviance residuals

Deviance residuals are derived based on the deviance, which is defined as twice the difference between the log-likelihood for the saturated and fitted models and is given by $D(y, \hat \mu)=2\sum _{i=1}^{n}\left \{{log} [p(y_{i}|\hat \theta _{s})]-{log} [p(y_{i}|\hat \theta)]\right \}$, where ${log} [p(y_{i}|\hat \theta _{s})]$ represents the log-likelihood function for the *saturated model* and *θ*_*s*_ denotes the set of parameter estimates for the saturated model, in which there are as many estimated parameters as data points [8, 18]. By definition, a saturated model leads to a perfect fit to the data and has the highest log-likelihood among all models. $\text {log}[p(y_{i}|\hat \theta)]$ represents the log-likelihood function of the fitted model and $\hat \theta $ denotes the set of parameter estimates for the fitted model. Deviance residuals represent the contributions of individual samples to the deviance $D(y, \hat \mu)$, which is defined as the signed square root of the corresponding component for $D(y, \hat \mu)$ and can be written as

8 $$ r^{D}_{i}=\text{sign}(y_{i}-\hat{\mu}_i)\sqrt{d_{i}},  $$

where $d_{i}=2 \left \{\text {log} [p(y_{i}|\hat \theta _{s})]-\text {log} [p(y_{i}|\hat \theta ])\right \}$. For a ZIP model, it can be shown that Poisson(*y*_*i*_) is the saturated model [19]; hence, the deviance residual for a ZIP model is defined as the signed square root of the likelihood ratio between the fitted model (zero-inflated Poisson model) and the saturated model (Poisson model). The deviance residuals for a ZINB model can be defined similarly. Table 2 presents the deviance residuals for some commonly used count regression models.

**Table 2:** Deviance residuals for commonly used regression models for count data

Model	Deviance residuals
Poisson	$r^{D}_{i} ={sign}(y_{i}-\hat {\lambda _{i}}) \left \{2\left [ y_{i} \log \frac { y_{i}}{\hat {\lambda _{i}}} - (y_{i} - \hat {\lambda _{i}}) \right ] \right \}^{1/2} $
NB	$r^{D}_{i} = {sign}(y_{i}-\hat \lambda _{i})\left \{2\left [y_{i} \log \frac { y_{i}}{\hat \lambda _{i}} - (y_{i} +k)\log \frac {y_{i}+k}{\hat \lambda _{i}+k} \right ] \right \}^{1/2} $
ZIP	$ r^{D}_{i} = {sign}(y_{i}-\hat {\mu }_{i}) \left (2\left \{ -y_{i} +y_{i} \log y_{i} -\log y_{i} !\right.\right. $
	$\quad \quad \quad \quad \quad \quad \quad \quad \quad \quad \quad \quad \quad \quad \ \ - I(y_{i}=0)\log \left [ \hat {p}_{i} +(1-\hat {p}_{i}) e^{-\hat {\lambda }_i} \right ] $
	$\quad \quad \quad \quad \quad \quad \quad \quad \quad \quad \quad \quad \quad \quad \ \left.\left. - I(y_{i} > 0)\left [\log (1-\hat {p}_{i}) - \hat {\lambda }_{i} +y_{i} \log \hat {\lambda }_{i} -\log y_{i} ! \right ] \right \}\right)^{1/2} $
ZINB	$r^{D}_{i} ={sign}(y_{i}-\hat {\mu }_i) \left (2\left \{{log}\frac {\Gamma (y_{i}+k)}{\Gamma (k)\Gamma (y_{i}+1)} +y_{i} {log}\left (\frac {y_{i}}{y_{i}+k}\right)+k{log}\left (\frac {k}{y_{i}+k}\right)\right.\right.$
	$\quad \quad -I(y_{i}=0) {log} \left [p_{i}+(1-p_{i})\left (\frac {k}{\lambda _{i}+k}\right)^{k}\right ]$
	$\quad \ \ \left.\left.-I(y_{i}>0) \left [{log}(1-p_{i})+{log}\frac {\Gamma (y_{i}+k)}{\Gamma (k)\Gamma (y_{i}+1)} +y_{i} {log}\left (\frac {y_{i}}{y_{i}+k}\right)+k{log}\left (\frac {k}{y_{i}+k}\right)\right ] \right \}\right)^{1/2} $

### Problems with pearson and deviance residuals

For a normal linear regression model, the Pearson and deviance residuals are identical and have an approximate normal distribution under the true model. However, their distributions are often skewed and non-normally distributed for counts regression models [8, 20]. It is argued that the deviance residuals typically follow more closely a normal distribution than the Pearson residuals; nevertheless, as *μ*_*i*_/*ϕ*→*∞*, both Pearson and deviance residuals from an exponential family model approach to the normal distribution due to the distribution for the response variable converging to normality. However, the asymptotic normal distribution only holds when the mean of the response variable is relatively large. Further, the residual plots often exhibit parallel curves according to distinct response values, imposing great challenges for visual inspection. Hence, Pearson and deviance residuals are difficult to use for graphically assessing the GOF of count regression models.

Further, the overall GOF of a regression model is often assessed based on the sum squares of the Pearson and deviance residuals, i.e., $ X^{2}=\sum _{i=1}^{n} r^{P^{2}}_{i}$ and $D^{2}=\sum _{i=1}^{n} r^{D^{2}}_{i}$, respectively. Asymptotically, under a correctly specified normal regression model, we can expect *X*^2^ and *D*^2^ to have a chi-square distribution $\chi ^{2}_{n-p}$, where *n* is the sample size, and *p* is the number of parameters. In practice, we often fail to achieve large samples, which renders the null distribution of this statistic invalid. It is also recognized that this approximation for diagnosing count regression models can be very poor even for large sample sizes [9, 21].

### Randomized quantile residual

#### Definition of randomized quantile residuals

Randomized quantile residual (RQR) proposed by Dunn and Smyth [10] gives a general tool for diagnosing regression models with independent responses, which is particularly ideal for diagnosing count regression models. RQR is an extension of quantile residuals, which is based on the idea of inverting the estimated distribution function for each observation to obtain approximately normally distributed residuals.

In the case of a normal regression model, quantile residuals are equivalent to Pearson and deviance residuals [14]. In the case of a non-normal regression model for modeling a highly skewed and continuous outcome variable, Scudilio and Pereira (2020) [14] proposed an adjusted quantile residual to diagnose inverse Gaussian or Gamma regression models, which was shown to be a better choice to perform diagnostic analysis compared to traditional residuals. Pereira (2019) [12] and Arenas (2019) [13] investigated the properties of the quantile residual in the beta regression and generalized Johnson *S*_*B*_ regression models, respectively. Their results showed that the quantile residual is well approximated by a standard normal distribution.

In the case of the discrete outcome, such as Poisson or NB, the corresponding CDFs are discrete. To circumvent this issue, the RQR introduces randomization between two consecutive CDFs to produce continuous normal residuals [10]. More specifically, suppose we consider fitting a regression model with $F(y_{i};\hat \mu _{i}, \hat \phi)$ denoting the CDF for a response variable *y*_*i*_ given a set of covariates *x*_*i*_, where $\hat \mu _{i}$ is typically a function of *x*_*i*_ (i.e., the conditional mean of *y*_*i*_) and *ϕ* (i.e., the dispersion parameter) does not depend on *x*_*i*_. Let $p(y_{i}; \hat \mu _{i}, \hat \phi)$ be the corresponding PMF of $F(y_{i}; \hat \mu _{i}, \hat \phi)$. The estimated CDF is defined as

9 $$ F^{\ast} (y_{i}, u_{i};\hat\mu_{i},\hat\phi) = F(y_{i}-; \hat\mu_{i},\hat\phi)+u_{i} \cdot \, p(y_{i};\hat\mu_{i},\hat\phi),  $$

where $F(y_{i}-;\hat \mu _{i},\hat \phi)$ is the lower limit of *F* at *y*_*i*_ (i.e., $\sup _{y < y_{i}} F(y;\hat \mu _{i})$) and *u*_*i*_ is a random number from a uniform distribution on (0,1). An alternative definition for the randomized lower tail probability is a uniform random number between $a=\sup _{y < y_{i}} F(y;\hat \mu _{i}, \hat \phi)$ and $b = F(y_{i}; \hat \mu _{i}, \hat \phi)$ [10].

RQR for *y*_*i*_ is then calculated as the standard normal quantile of the estimated CDF, which is given by

10 $$ z^{Q}_{i} = \Phi^{-1}[F^{\ast} (y_{i}, u_{i};\hat{\mu}_i,\hat{\phi})],  $$

where *Φ*^−1^ is the quantile function of the standard normal distribution. $F^{\ast } (y_{i}, u_{i};\hat {\mu }_i,\hat {\phi })$ can be converted to any other previously mentioned standard distribution. The normal distribution is chosen due to its well-known characteristic (i.e., the so-called “empirical rules”), and diagnostic and inspection methods for normal linear regression can be then applied to RQRs. Note that when *F* is continuous at *y*_*i*_, in Eq. (10), PMF at *y*_*i*_ is $p(y_{i};\hat \mu _{i},\hat \phi) = 0$; for clarification, $p(y_{i};\hat \mu _{i},\hat \phi) $ is not the PDF of *y*_*i*_. Therefore, there is no actual “randomness” in $F^{\ast } (y_{i}, u_{i};\hat \mu _{i},\hat \phi)$ when response distribution is continuous. The formula given in (9) encompasses this situation. Therefore, in the case of continuous response variable, RQRs are equivalent to quantile residuals. Similarly, when *y*_*i*_ is discrete, the variability in *F*^∗^ tends to be smaller for narrower gaps at the discontinuity points of *F*. This scenario typically occurs when the mean of response variable is large.

Klar and Meintanis (2012) [11] suggested standardizing randomized quantile residuals, which are calculated as $r^{Q}_{i}=\frac {z^{Q}_{i}-\bar {z}^{Q}}{S_{z^{Q}}}$, where $\bar {z}^{Q}=n^{-1}\sum _{i=1}^{n} z^{Q}_{i}$ and $S^{2}_{z^{Q}}=(n-1)^{-1}\sum _{i=1}^{n}\left (z^{Q}_{i}-\bar {z}^{Q}\right)^{2}$ for performing goodness-of-fit tests of common generalized linear models including Poisson, NB, Gamma and Inverse Gaussian. The standardized RQRs were shown to approximately follow a standard normal distribution. Following their suggestion, we standardized RQRs in our investigation. In this paper, we show that RQRs can also be applied for examining the model fits of count models in general, including zero-inflated models. Table 3 provides the derivation of the RQRs for the Poisson, NB, and ZIP and ZINB regression models, where *ppois* and *dpois*, *pnb* and *dnb*, *pzip*, *dzip* and *pzinb* and *dzinb* denote their respective CDFs and PMFs.

**Table 3:** RQRs for commmonly used regression models for count data

Model	RQRs
Poisson	$z^{Q}_{i} = \Phi ^{-1} \left (ppois(y_{i}-1;\hat {\mu }_i)+u_{i} \cdot dpois(y_{i};\hat {\mu }_i) \right)$
NB	$z^{Q}_{i} = \Phi ^{-1} \left (pnb(y_{i}-1;\hat {\mu }_i, \hat {k})+u_{i} \cdot dnb(y_{i};\hat {\mu }_i,\hat {k}) \right)$
ZIP	$z^{Q}_{i} = \Phi ^{-1} \left (pzip(y_{i}-1;\hat {\mu }_i,\hat {p_{i}})+u_{i} \cdot dzip(y_{i};\hat {\mu }_i,\hat {p_{i}})\right)$
ZINB	$z^{Q}_{i} = \Phi ^{-1} \left (pzinb(y_{i}-1;\hat {\mu _{i}},\hat {k},\hat { p_{i}})+u_{i} \cdot dzinb(y_{i};\hat {\mu _{i}},\hat {k},\hat { p_{i}})\right)$

The idea of constructing *F*^∗^ in (9) is also closely related to the predictive *p*-values proposed for diagnosing Bayesian models for discrete observations in which the *u*_*i*_ is fixed at 0.5 rather than being a uniform random number (See [22–25]). This approach creates non-random quantile residuals, to which we refer as “middle-point quantile residuals (MQRs)”, which will also be compared with the RQRs in this study.

#### An illustrative example

To demonstrate the idea of the RQRs, we simulate a response variable of size *n*=1000 from a Poisson model with log(*μ*_*i*_)=−1+2sin(2*x*_*i*_), where *μ*_*i*_ is the expected mean count for the *i*th subject and *x*_*i*_∼*Uniform*(0,2*π*), *i*=1,…,*n*. Then, we fit a Poisson model with the same mean structure as well as a Poisson model with a misspecified mean structure log(*μ*_*i*_)=*β*_0_+*β*_1_*x*_*i*_ to illustrate the capability of RQRs for detecting the non-linearity of the covariate effect.

**Fig. 1:**
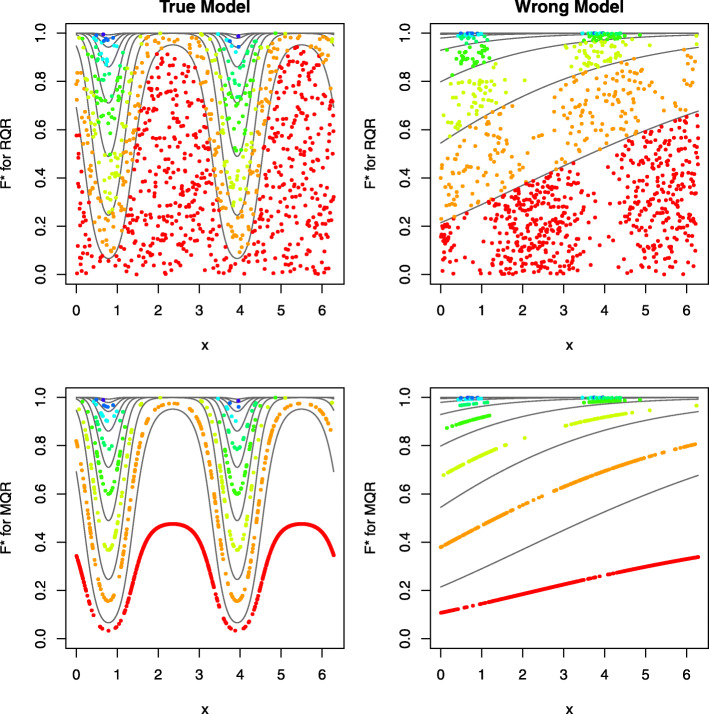
An illustrative example of the estimated CDFs, *F*^∗^, under RQRs (top panels) and MQR (bottom panels). Panels in the left column present the residuals under the true model and panels in the right column present the residuals under the wrong model. The curved grey lines correspond to the theoretical CDF of *F*(*k*|*x*_*i*_)versus*x*_*i*_ at each value of *k*. The points are the randomized CDFs $F^{\ast } (y_{i};\hat {\mu }_i,\hat {\phi }, u_{i})$ with each colour corresponding to a unique value of *y*_*i*_

The CDF of the response variable *Y*_*i*_ given *x*_*i*_ (under the considered model with estimated parameters) is denoted by *F*(*k*|*x*_*i*_)=*P*(*Y*_*i*_≤*k*|*x*_*i*_), for *k*=0,1,…. Figure 1 shows *F*(*k*|*x*_*i*_) as a function of *x*_*i*_, with each coloured line representing a CDF curve associated with a value *k*. The distance between two curves, *F*(*k*|*x*_*i*_) and *F*(*k*−1|*x*_*i*_), is the “theoretical” (model-based) probability of *y*_*i*_=*k* given each *x*_*i*_. Each observed *y*_*i*_ is then randomly scattered uniformly to a point between the CDF lines associated with *k*=*y*_*i*_−1 and *k*=*y*_*i*_. The pattern of randomized scattering of discrete *y*_*i*_ facilitates the comparison of the “observed” frequency (fraction of points) and “theoretical” frequency (distance of two lines). If the “observed” and “theoretical” frequencies agree well, the randomly scattered points of $F^{\ast } (y_{i};\hat {\mu }_i,\hat {\phi }, u_{i})$ should be uniformly distributed on (0,1] in each neighbourhood of *x*_*i*_. Figure 1 depicts that under the true model, the randomized CDFs are uniformly distributed on (0, 1] given each *x*_*i*_. By contrast, under the misspecified model, the randomized CDFs are not uniformly distributed, exhibiting a non-linear trend. The results also show that MQRs have the same difficulties as the Pearson and deviance residuals for model assessment with residuals clustering along curved lines for both true and wrong models.

**Fig. 2:**
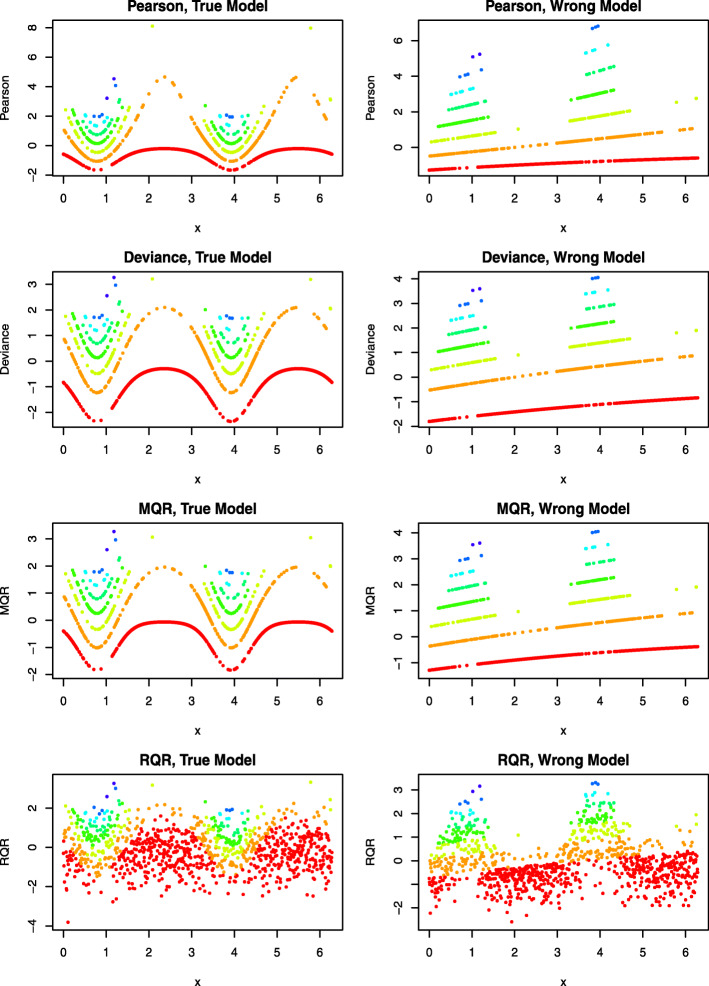
An illustrative example of the RQRs in comparison with other residuals. Panels in the left column present the residuals under the true model and panels in the right column present the residuals under the wrong model

Figure 2 displays the scatter plots of various types of residuals against the covariate *x* under the true and wrong models, which show that the Pearson, deviance, and MQRs are clustered as curves according to the distinct and discrete values of the response variable. By contrast, under the true model, RQRs are randomly scattered between -3 and 3; under the wrong model, the plot of RQRs against *x* exhibits a sinusoidal trend.

To examine whether standardized RQR is well approximated by a standard normal distribution under the true model as compared with other types of residauls, Table 4 reports the mean, variance, skewness, excess kurtosis for the Pearson residual, deviance residual, MQR and RQR, under the true and wrong models, respectively. The skewness is a measure of symmetry. Negative skewness indicates that the data is left-skewed and positive skewness indicates that the data is right-skewed. The excess kurtosis describes the tail shape of the data distribution, so a normal distribution has zero excess kurtosis. Negative excess kurtosis would indicate a thin-tailed data distribution, and positive excess kurtosis indicates a fat-tailed distribution. We also used Shapiro-Wilk (SW) test [26] to evaluate the normality of the residuals with the null hypothesis *H*_0_: The residuals are normally distributed versus the alternative hypothesis *H*_*a*_: The residuals are not normally distributed. Previous research showed that SW test is more powerful compared to other normality tests, such as Kolmogorov–Smirnov test, the Lilliefors test, the Cramer–von Mises test, the Anderson–Darling test, the D’Agostino–Pearson test, etc., over a wide range of asymmetric distributions [27].

**Table 4:** Mean, variance, skewness, kurtosis and the *p*-value of SW normality test for the Pearson residual, deviance residual, MQR and RQR in the illustrative example

	Model	Pearson	Deviance	MQR	RQR
Mean	True	0.003	-0.213	0.072	0.003
	Wrong	-0.002	-0.336	-0.014	-0.067
Variance	True	1.012	0.809	0.597	1.000
	Wrong	1.993	1.592	1.154	1.000
Skewness	True	1.828	0.646	0.756	-0.015
	Wrong	1.990	1.171	1.400	0.744
Kurtosis	True	4.609	0.572	1.060	0.205
	Wrong	4.162	0.630	1.464	0.499
SW test	True	0.000	0.000	0.000	0.684
	Wrong	0.000	0.000	0.000	0.000

The results presented in Table 4 indicate that the means of the Pearson residuals, MQRs and RQRs are all close to zero, but the means of the deviance residuals are below zero under both the true and wrong models. This finding is consistent with the previous investigations [13, 14], which showed the deviance residuals do not follow a standard normal distribution regardless of the true or wrong models. The variances of Pearson residuals under both the true and wrong models are above one. Under the true model, the variances of the deviance residuals and MQRs are all below one, but above one under the wrong model. By contrast, the variances of RQR are equal to one for all models, since the RQRs in the present study are all standardized to have unit variance. In terms of skewness and kurtosis, the Pearson residual, deviance residual, and MQR are right-skewed and heavy-tailed relative to a normal distribution, and the extent of skewness and heavy-tailed is more pronounced for Pearson residual. RQRs are approximately symmetric with the tail shape close to a normal distribution under the true model and are right-skewed under the wrong model. For testing the normality of the residuals based on the SW normality test, the results clearly showed that both true and wrong models were rejected according to the Pearson residual, deviance residual, and MQR. By contrast, RQRs confirm the adequacy of the true model with the *p*-value of the SW normality test equal to 0.684 and inadequacy of the wrong model with the SW *p*-value almost equal to zero.

**Fig. 3:**
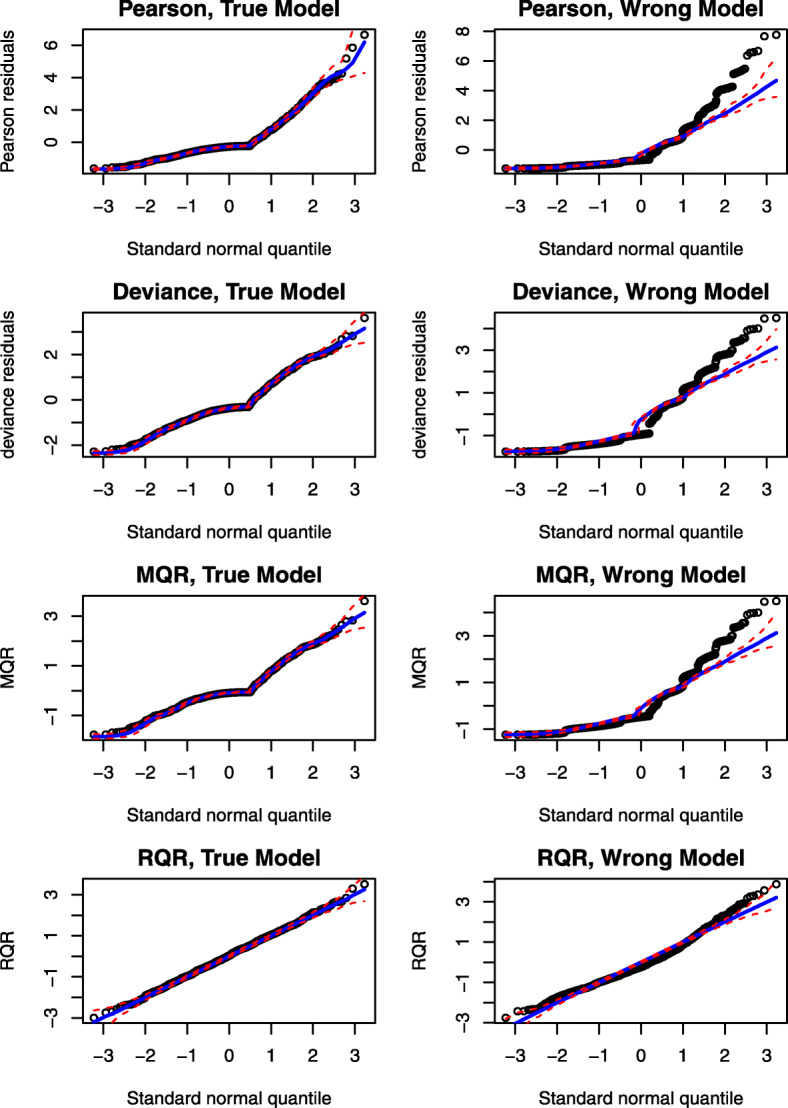
An illustrative example of the normality QQ plots of RQRs in comparison with other residuals. Panels in the left column present QQ plots for the residuals under the true model, and panels in the right column present the QQ plots for the residuals under the wrong model. The red dashed lines represent the simulated envelope

Further, normal quantile-quantile (QQ) plots can be produced by plotting the ordered values of the residuals versus the expected order statistics of a normal distribution, approximated as $\Phi ^{-1}(\frac {i-3/8}{n+1/4})$, where *i* is the *i*th order statistic, 1≤*i*≤*n* and *n* is the sample size [28]. If the residuals are normally distributed, the points in the QQ plot should follow a straight diagonal line. However, for the count regression models, the asymptotic distributions of the residuals are unknown; therefore, the normal QQ plot is not informative for diagnosing model fits of count regression models. To overcome this challenge, a simulated envelope proposed by Atkinson (1981) [29] can be added to the QQ plot to detect departures from the distributional assumptions as well as outlying observations of the fitted model. The simulated envelope can be obtained as follows [30]: (1) fitting a model, (2) extracting and sorting the residuals, (3) simulating 100 response variable using the same model matrix, error distribution and parameter estimates, (4) fitting the same model to each simulated response variable and extracting and sorting the residuals, and (5) computing the 2.5% and 97.5% percentiles of the simulated residuals at each ordered residuals to form the envelope. For a well-fitted model, most of the residuals are expected to fall within the simulated envelope. As displayed in Fig. 3, all types of residuals under the true model fall within the simulated envelopes. By contrast, under the wrong model, a bulk of residuals fall outside of the simulated envelopes.

The results indicate that the normality probability plot with a simulated envelope can help distinguish the true and wrong models; however, visualizing these plots could not provide information on the nature of deficiencies with respect to the fitted model. In contrast, the scatter plot of RQRs against covariates or fitted values can provide information on the inadequacy of different aspects of the models, such as the misspecified functional form of the covariate effect, over-dispersion, and zero-inflation, etc. Moreover, a numerical measure of overall model goodness of fit is desirable to summarize the discrepancy between fitted and observed values under the fitted model rather than only relying on visualizing the QQ plots with simulated envelope to determine the model fit, which can be subjective.

## Results

### Simulation studies

In this Section, we investigate the performance of the RQRs in comparison with MQRs, deviance, and Pearson residuals via simulations. The simulations consist of testing non-linearity of the covariate effect, over-dispersion, and zero-inflation. A numerical measure of overall model goodness of fit is desirable to summarize the discrepancy between expected values under the fitted model and observed. It is also desired to have a unifying framework for all types of models using residuals that are approximately standard normally distributed in line with a normal regression model. We, therefore, use the SW test evaluating the normality of residuals as a goodness-of-fit test with the null hypothesis *H*_0_: The model fits the data well versus the alternative hypothesis *H*_*a*_: The model does not fit the data well.

Under each simulation scenario, we randomly generated 5000 datasets from the true model to examine the type I error rate and statistical power. The type I error rate is defined as the probability of rejecting the true model (i.e., the proportion of times that the SW test *p*-value <5*%* for the residuals under the true model) and statistical power is the probability of rejecting the wrong model (i.e., the proportion of times that the SW test *p*-value <5*%* for the residuals under the wrong model). Ideally, a desirable GOF test should have a probability of type I error close to 5% with high statistical power.

For each simulation, we first present the performance of RQR for detecting model misspecification under one simulation scenario. Further, to gain more insight into the finite-sample performance of RQRs in comparison with other residuals, we perform power analysis by setting the sample sizes to *n*=20,50,100,200,400,600,800 and 1000 with varying degrees of model misspecification.

#### Detection of non-linearity

In this subsection, we first investigate the performance of the RQRs for detecting non-linearity of the covariate effect based on a single simulation setting. We first simulate the covariate *x*∼*Uniform*(−1.5,1.5) of size *n*=1000. The response variable is then simulated from a NB regression model $\text {log}(\mu _{i})=\beta _{0}+\beta _{1} x_{i}^{2}$, where *μ*_*i*_ is the expected mean count for the *i*th subject. We then consider fitting the model assuming *x*_*i*_ having a linear effect, i.e., log(*μ*_*i*_)=*β*_0_+*β*_1_*x*_*i*_. The regression parameters were set as *β*_0_=0 and *β*_1_=1 while the reciprocal for the dispersion parameter associated with the NB distribution was set as 2.

**Fig. 4:**
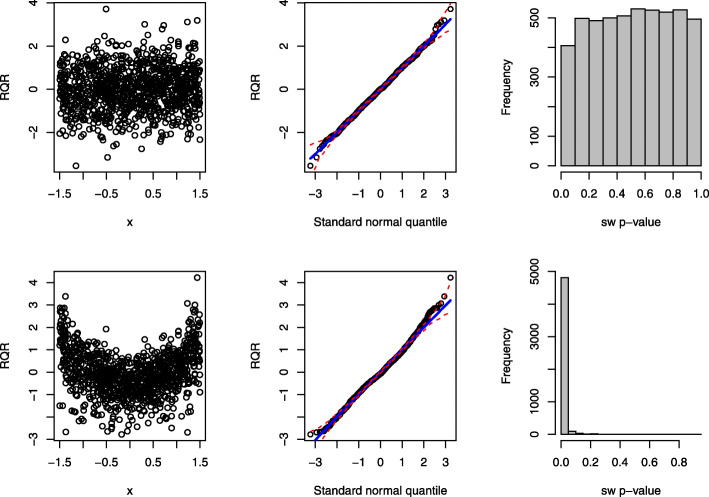
Performance of the RQRs in detecting covariate non-linearity effect of a sample dataset of size *n*=1000. The panels in the first row present the RQRs for the true fitted model: NB model with quadratic covariate effect (i.e., exp(*β*_1_*x*^2^)). The panels in the second row present the RQRs for the fitted wrong model: NB model with linearity covariate effect (i.e., exp(*β*_1_*x*)). The first two columns display the scatter plots and QQ plots of the RQRs, respectively. The red dashed lines in the QQ plots represent the simulated envelopes. The third column presents the histograms of the SW *p*-values for the RQRs over 5000 randomly generated datasets from the true model

The panels in the first column of Fig. 4 display the scatter plots of RQRs against the covariate under the true and wrong models. Under the true model, RQRs are randomly scattered without exhibiting any pattern and being mostly distributed between -3 and 3 as standard normal variates; conversely, under the wrong model, the RQRs clearly show a quadratic pattern. The panels of the second column of Fig. 4 present the quantile-quantile (QQ) plots of the RQRs under the true and wrong models. Under the true model, the points almost perfectly align along the diagonal line, whereas under the wrong model, the points deviate from the diagonal line in both upper and lower ends with some of the points on the ends falling outside of the simulated envelope.

To examine the power of the SW test for RQRs as an overall GOF, we repeatedly simulated 5000 datasets from the true model. The panels in the third column of Fig. 4 present the histograms of 5000 SW *p*-values under the true and wrong models. The SW *p*-values under the true model are nearly uniform, indicating the well-calibration of this overall GOF test. In contrast, under the wrong model, the SW *p*-values are highly distributed near 0. Thus, the SW test for the RQRs as an overall GOF test seems to perform well with type I error close to 5% and great power in detecting the non-linear relationship in this simulation setting.

**Fig. 5:**
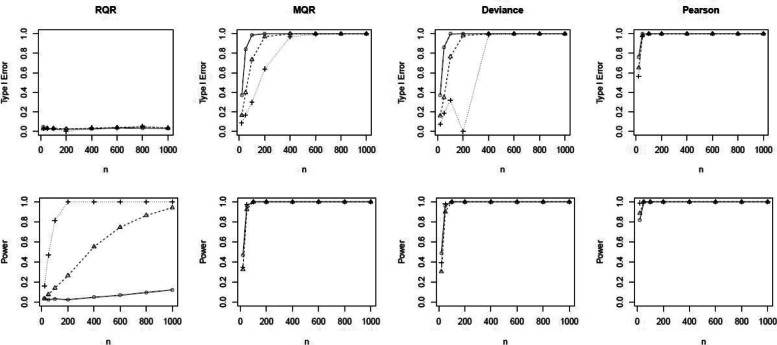
Comparison of the type I error and power of the SW tests for RQR, MQR, deviance residual and Pearson residual. Response variable is simulated from the true model at varying sample sizes of *n*=20,50,100,200,400,600,800 and 1000, and nonlinear covariate effects of *β*_1_=0.5 (), 1 () and 2 (). True model: NB model with mean exp(*β*_1_*x*^2^). Wrong model: NB model with mean exp(*β*_1_*x*)

To further investigate the performance of the SW test for RQRs as an overall GOF test, we conducted the power analysis by setting *β*_1_=0.5,1,2 to increase the degree of non-linearity of the covariate effect. As shown in the top panels of Fig. 5, type I errors of the SW test for RQRs are consistently centered around the 5% nominal level for all scenarios. In contrast, type I errors for the SW tests for MQRs, deviance, and Pearson residuals approach to 1 as sample size increases. The bottom panels of Fig. 5 indicate that the power of this GOF test based on RQRs increases as the sample size increases, especially when the misspecified model deviates from the true model at a greater degree. Despite the high power of the SW tests based on MQRs, deviance, and Pearson residuals, they suffer from substantially high type I errors. As a result, overall GOF tests based on SW tests of MQRs, deviance, or Pearson residuals are undesirable in this scenario.

#### Detection of over-dispersion

As in the previous section, the same approach is implemented to investigate the performance of the RQRs in detecting over-dispersion [31] in the data. We first simulate a covariate *x*∼*Uniform*(−1,2) of size *n*=1000. Then, the response variable is simulated from a NB model *log*(*μ*_*i*_)=*β*_0_+*β*_1_*x*_*i*_, where *μ*_*i*_ is the expected mean count for the *i*th study subject. We set the regression parameters as *β*_0_=1 and *β*_1_=2 and reciprocal for the dispersion parameter as 2. To examine the performance of the various types of residuals in diagnosing over-dispersion, we considered fitting a Poisson model as the misspecified model, which has the same mean structure as the NB model.

**Fig. 6:**
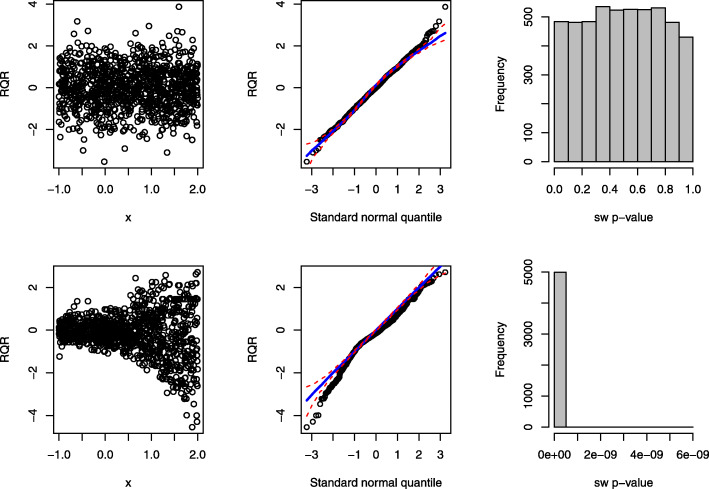
Performance of the RQRs in detecting over-dispersion of a sample dataset of size *n*=1000. The panels in the first row present the RQRs for the true fitted model: NB model. The panels in the second row present the RQRs for the fitted wrong model: Poisson model with the same mean structure as the true model. The first two columns display the scatter plots and QQ plots of the RQRs, respectively. The red dashed lines in the QQ plots represent the simulated envelopes. The third column presents the histograms of the SW *p*-values for the RQRs over 5000 randomly generated datasets from the true model

The panels in the first column of Fig. 6 present the scatter plots of the RQRs against the covariate under the true and wrong models. Under the true model, the residuals are mostly scattered between -3 and 3 without any discerning pattern. In contrast, under the wrong model, the residuals “fan out” from left to right, suggesting the presence of over-dispersion at increasing values of *x*_*i*_. The panels in the second column of Fig. 6 present the QQ plots of RQR residuals under the true and wrong models. Under the true model, the points align along the diagonal line well; whereas, under the wrong model, the points significantly deviate from the diagonal line with a substantial amount of the points falling outside of the simulated envelope, indicating the RQRs are approximately normally distributed under the true model, but not under the wrong model. The panels in the third column of Fig. 6 present the histograms of 5000 SW *p*-values for testing the normality of the RQRs under the true and wrong models. As shown, the SW *p*-values under the true model are nearly uniform, while the SW *p*-values under the wrong model are clustered around 0. These results demonstrate that the SW test for the RQRs as an overall GOF test can help detect over-dispersion.

**Fig. 7:**
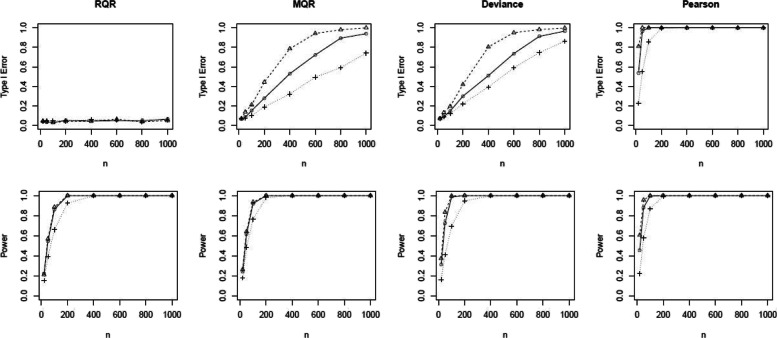
Comparison of the type I error and power of the SW tests for RQR, MQR, deviance residual and Pearson residual. Response variable is simulated from the true model at varying sample size, *n*=20,50,100,200,400,600,800,1000 and the over-dispersion parameter of 1 (), 2 () and 10 (). True model: NB model with mean exp(*β*_0_+*β*_1_*x*). Wrong model: Poisson model with mean exp(*β*_0_+*β*_1_*x*)

In the power analysis, we increased the level of over-dispersion in the data by setting the dispersion parameter as 1,2, and 10. Figure 7 shows that the type I errors of the SW test for the RQRs remain at the nominal level 0.05 for all scenarios. In contrast, the type I errors of the SW tests for the MQRs, deviance and Pearson residuals exceed 5% as sample size increases. Further, SW tests of the RQRs are able to maintain high statistical power at all scenarios when the sample size is sufficiently large (i.e., *n*>100).

#### Detection of zero-Inflation

Finally, we conduct simulations to investigate the performance of the RQRs in detecting zero-inflation. We first simulate a covariate *x*∼*Uniform*(−1,2) of size *n*=1000. Then, the response variable is derived from a ZIP model, where the expected mean for the Poisson component is *λ*_*i*_= exp(*β*_0_+*β*_1_*x*_*i*_). Under the true model, we set the regression parameters as *β*_0_=1 and *β*_1_=2, and the percentage of excessive zeros as 30%. A Poisson model with the same expected mean *λ*_*i*_, but ignoring zero-inflation is considered as the misspecified model.

**Fig. 8:**
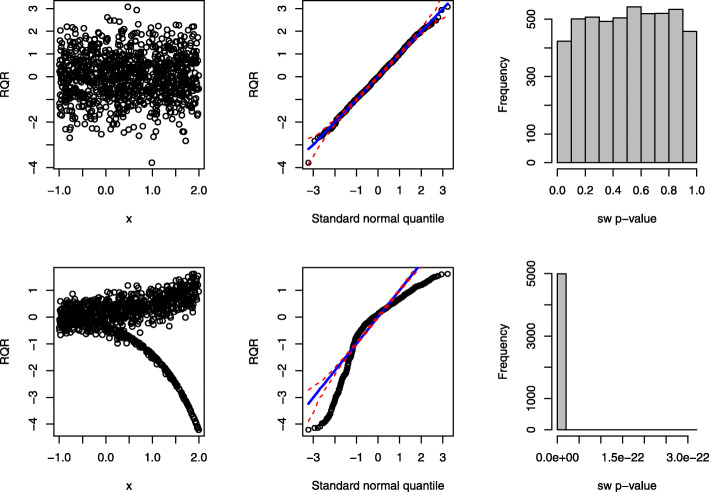
Performance of the RQRs in detecting zero-inflation of a sample dataset of size *n*=1000. The panels in the first row present the RQRs for the true fitted model: ZIP model. The panels in the second row present the RQRs for the fitted wrong model: Poisson model with the same mean structure as the true model. The first two columns display the scatter plots and QQ plots of the RQRs, respectively. The red dashed lines in the QQ plots represent the simulated envelopes. The third column presents the histograms of the SW *p*-values for the RQRs over 5000 randomly generated datasets from the true model

The panels in the top row of Fig. 8 display the scatter plot of RQRs against the covariate, QQ plot of RQRs, and histogram of 5000 SW *p*-values of the RQRs under the true model. The results indicate that RQRs are mostly distributed between -3 and 3 as standard normal variates without any unusual patterns. The points in the QQ plot align along the diagonal line, and the histogram of the SW *p*-values are nearly uniform. By contrast, under the misspecified model, a clear separation of the RQRs is observed from the residuals associated with the zero responses, as shown in the bottom left panel of Fig. 8. The points in the QQ plot under the misspecified model deviate from the diagonal line, with a substantial amount of the points falling outside of the simulated envelope. This histogram of SW *p*-values is highly distributed near 0, indicating that the wrong model was rejected most of the time. Hence, the SW tests for the RQRs as the overall GOF test can effectively detect zero-inflation.

**Fig. 9:**
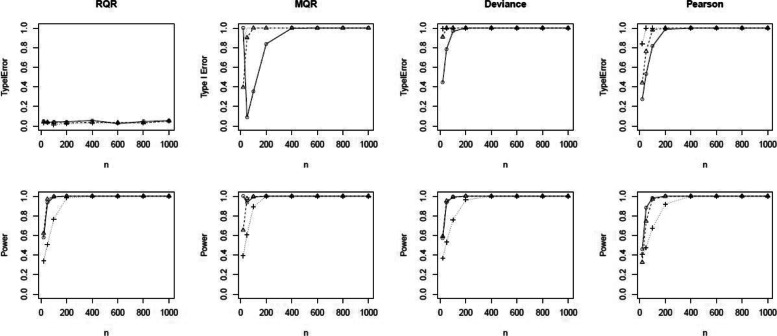
Comparison of the type I errors and powers of the SW tests for the RQR, MQR, deviance residual and Pearson residual. Response variable is simulated from the true model at varying sample size, *n*=20,50,100,200,400,600,800,1000 and percentage of excessive zeroes of *p*=10*%* (), 30% () and 50% (). True model: ZIP model with mean exp(*β*_0_+*β*_1_*x*). Wrong model: Poisson model with mean exp(*β*_0_+*β*_1_*x*)

In the power analysis, we set the probability of generating excessive zeros as 0.1, 0.3, and 0.5. Figure 9 shows that the type I error rates of the SW test for RQRs remain at the nominal level 5% for all scenarios. In contrast, the type I error rates of the MQRs, deviance, and Pearson yield extremely large type I error rates almost across all scenarios. In all scenarios, RQRs demonstrate descent statistical power even at small sample sizes, and the power increases as the sample size increases. Overall, RQRs outperform other types of residuals having low type I error and high power.

### Real data application

In this section, we examine the performance of RQRs in comparison with other types of residuals in a real data application based on the National Medical Expenditure Survey (NMES) (see further descriptions of this data in [32, 33]). In this data set, 4406 elderly in the United States were surveyed about their demands of health care. The response variable considered in this study is the number of emergency department (ED) visits. The covariates considered include demographic characteristics (e.g., age, race, sex, marital status, education, and region), socioeconomic variables (e.g., family income, employment status, supplementary private insurance status, and public insurance status) and health measures (e.g., self-perceived health, the number of chronic conditions and a measure of disability status).

Multicollinearity among all the covariates, was evaluated using the generalized variance inflation factor (GVIF) [34], which is a generalization of the variance inflation factor (VIF). GVIF is applicable to measure the collinearity among covariates, such as dummy regressors from a polytomous categorical variable, by considering the size of the joint confidence region for the related coefficients. Literature suggests reporting GVIF^1/(2·*df*)^, where *df* is the number of dummy variables in a categorical variable, which is analogous to reporting the square root of the VIF for a single coefficient [34]. As a rule of thumb, VIF of 5 (i.e., $\sqrt {{VIF}}\approx 2.236$) or greater indicates multicollinearity is high. Table 7 in Appendix reported no values of *GVIF*^1/(2·*df*)^ are greater than 2.236; therefore, all the covariates considered are not highly correlated in this analysis.

Over 81% of the patient-year records were zero, implying that the majority of patients did not make any ED visits during the year of the study. The number of non-zero visits ranged from 1 to 12, with only 5% having more than one visit in the study year. Given the high percentage of zeros and skewness of the response variable, we considered fitting Poisson, NB, ZIP, and ZINB regression models. After backward elimination at the 5% significance level, the final models included the following covariates: The number of chronic conditions, self-perceived health (excellent vs. poor; average vs. poor), limited activities of daily living (yes vs. no) and the number of years of education. In addition to those covariates, the black race was significantly associated with increased ED use for the Poisson and ZIP models, but not for the NB and ZINB models. This discrepancy highlighted the importance of examining the model GOF; that is, fitting a model with unsatisfactory model fit may lead to biased estimates, incorrect standard errors, and erroneous inferences.

For the binary components of the ZIP and ZINB models, no covariates were statistically significant at the 5% level by including one variable at a time or using backward selection. Table 5 presented the results of the analyses, which showed that the standard errors of the estimated regression coefficients for the NB and ZINB models are all larger relative to their counterpart Poisson models (i.e., Poisson and ZIP models), indicating that the choice of model distribution has a significant impact on covariate effects. NB and ZINB models yield almost identical estimated regression coefficients, with ZINB model giving slightly less efficient estimates compared to the NB model; that is, the standard errors for the estimated regression coefficients of chronic conditions and self-perceived health (Average vs. poor) were slightly larger under the ZINB model as compared to the corresponding standard errors under the NB model.

**Table 5:** Estimated regression coefficients and the standard errors (in parentheses) for the Poisson, NB, ZIP and ZINB models in the real data application

Variables	Poisson	NB	ZIP	ZINB
Black vs. others	0.188(0.085)*	−	0.300(0.097)*	−
Chronic conditions	0.221(0.020)**	0.217(0.026)**	0.216(0.023)**	0.217(0.027)**
Self-perceived health				
Excellent vs. poor	-1.093(0.190)**	-1.089(0.216)**	-1.028(0.207)**	-1.089(0.216)**
Average vs. poor	-0.505(0.074)**	-0.478(0.100)**	-0.451(0.088)**	-0.478(0.101)**
Limited activities	0.453(0.070)**	0.464(0.087)**	0.426(0.077)**	0.464(0.087)**
Years of education	-0.017(0.008)*	-0.023(0.010)*	-0.019(0.009)*	-0.023(0.100)*

^a^ Significance at the 5% and 1% level is indicated with ∗ and ∗∗, respectively

For comparing the competing models, the Akaike Information Criterion (AIC) is used with smaller values indicating better and more parsimonious model fit. The AIC scores for the Poisson, NB, ZIP, and ZINB are 5648, 5352, 5418, and 5354, respectively; this suggests that the NB and ZINB models are superior to their counterpart Poisson and ZIP models, respectively. NB model performs slightly better than the ZINB model. The intercept term for the binary component of the ZINB model is estimated as −8.912(SE: 41.106, *p*-value =0.828); therefore, the estimated probability of excessive zeros is about exp(−8.912)/[1+exp(−8.912)]≈0, which further confirms that the data does not exhibit significant more zeros than are expected under an NB distribution.

**Fig. 10:**
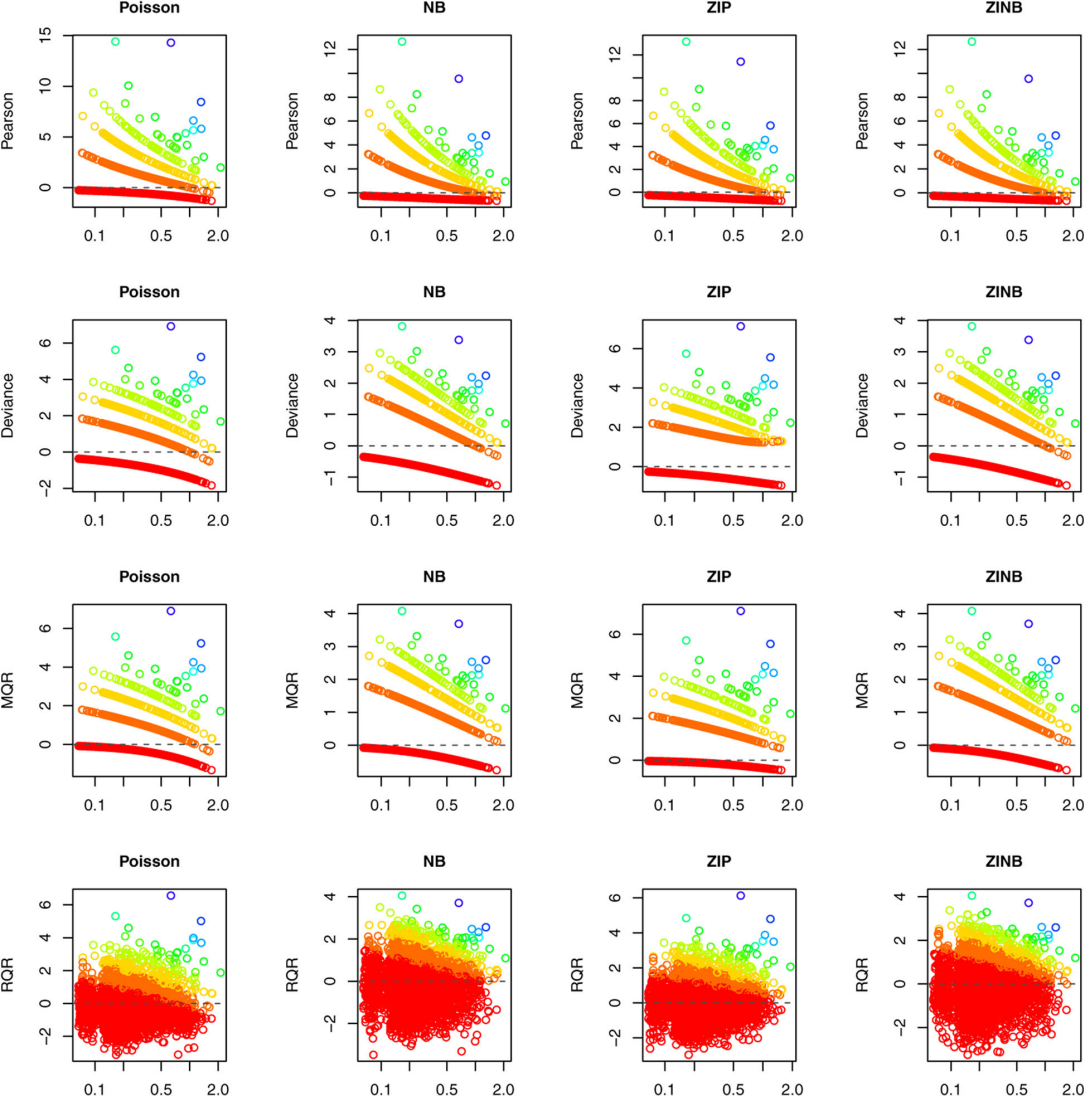
Scatter plots of the Pearson residual, deviance residual, MQR, and RQR versus fitted values under the Poisson, NB, ZIP, and ZINB models in the real data application modeling the number of ER visits. The rainbow colors correspond to the distinct values of the response variables ranging from red for the smallest value to blue for the largest value

**Fig. 11:**
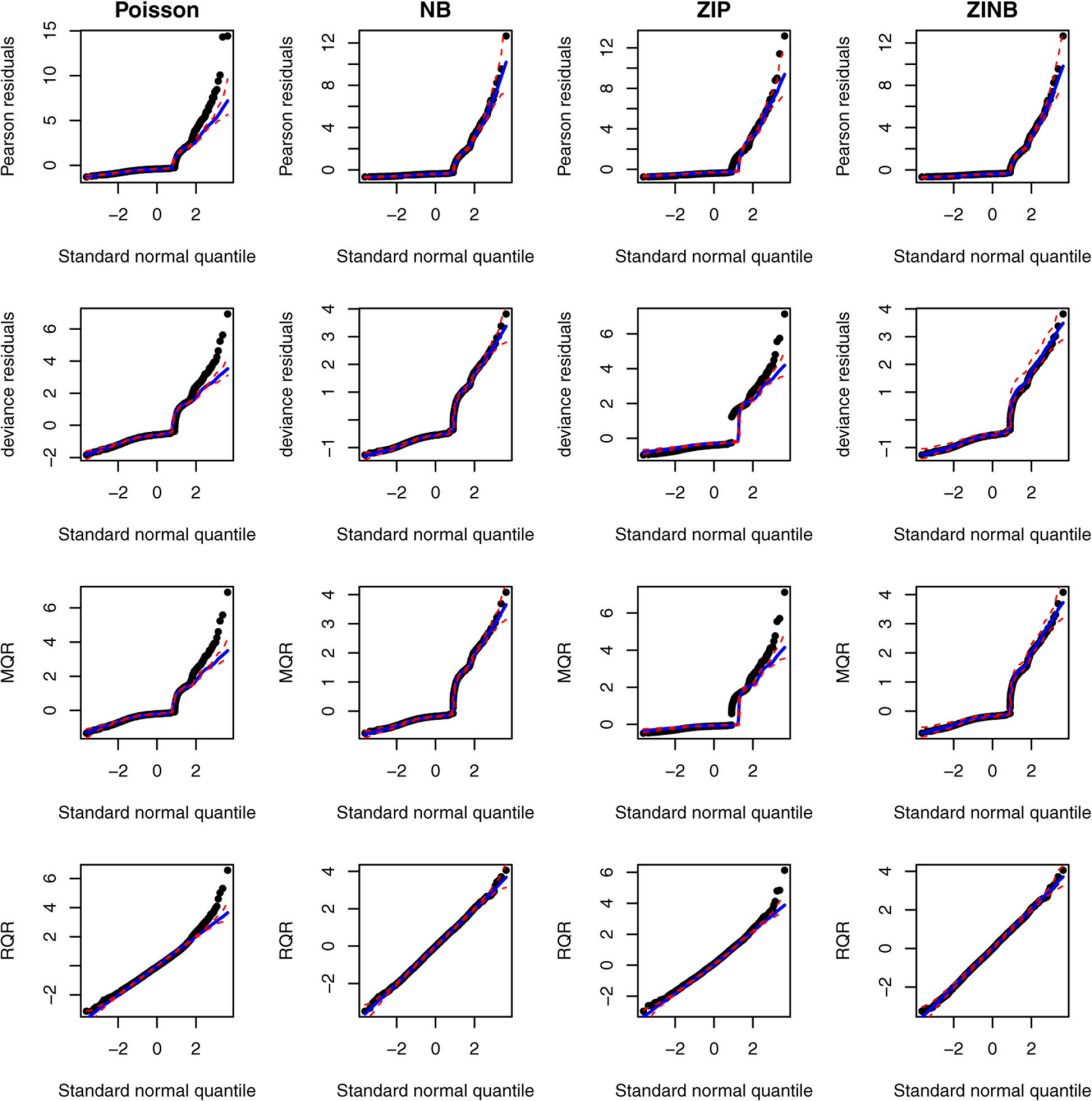
QQ plots for the Pearson residual (first row), deviance residual (second row), MQR (third row), and RQR (fourth row) under the Poisson, NB, ZIP and ZINB models in the real data application modelling the number of ER visits. The red dashed lines represent the simulated envelope

Although AIC can be used to compare the GOF of competing models, it cannot measure the adequacy of the model fit, assess the need for additional complexity, and validate the distribution assumption of the response variable. Diagnosing the residuals is, therefore, imperative to address these concerns. Figure 10 presented the scatter plots of Pearson, deviance, MQR, and RQRs versus fitted values under the Poisson, NB, ZIP, and ZINB models for modeling the number of ER visits. It is evident that Pearson, deviance, and MQR are clustered as curved lines. RQRs achieved continuity correction via randomization of the cumulative distribution function. The NB and ZINB models fitted the data fairly well with residuals ranging mostly between -3 and 3, and no discernible pattern. By contrast, Poisson and ZIP models do not appear to accommodate larger values of the response variable. The QQ plots of all types of residuals are presented in Fig. 11, which indicates that under the NB and ZINB models, the residuals fall within the simulated envelope. By comparison, under Poisson and ZIP models, a portion of residuals fall outside of the simulated envelopes. This indicates that NB and ZINB models fit the data adequately well, and Poisson and ZIP do not fit the data well, which further supports the need to model over-dispersion in this data.

**Fig. 12:**
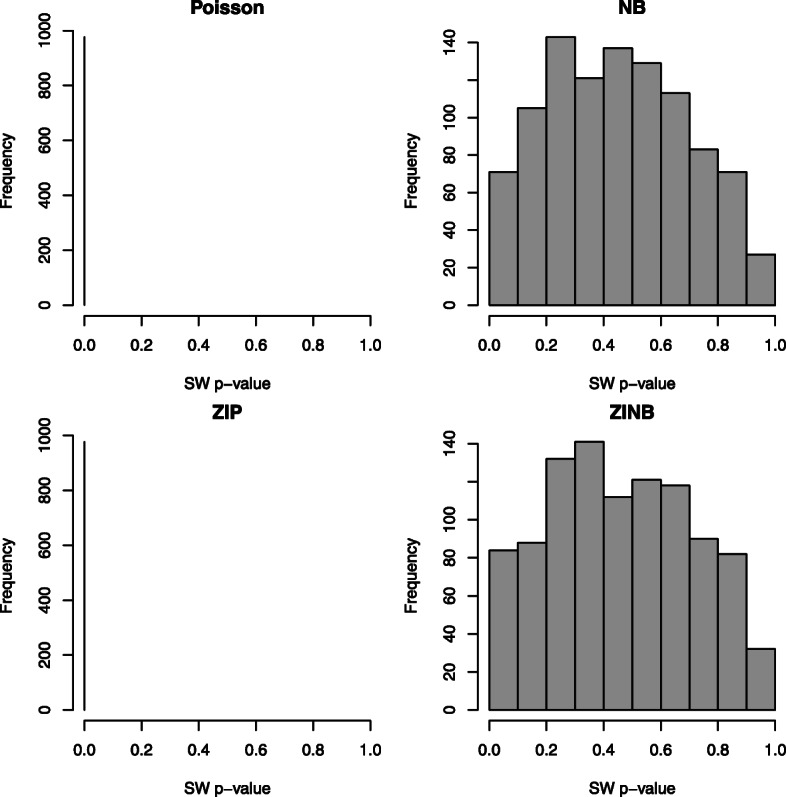
Histograms for the *p*-values of the SW normality test for 1000 replicated RQRs in the real data application

One concern of using RQRs is the fluctuation in the residuals introduced by randomization for producing continuously distributed residuals. As suggested by Dunn and Smyth [10], multiple realizations of the RQRs should be produced to ensure that the discrepancies are not made by the randomization in producing the residuals. To assess the uncertainty in the GOF test due to randomization in the real data application, we generated 1000 realizations of the RQRs. Figure 12 presents the histograms of 1000 replicated *p*-values of the SW tests, which indicates that randomization introduced little variation to the SW *p*-values of the RQRs in this application. More specifically, the SW *p*-values of RQRs under the fitted Poisson and ZIP models were close to 0, as depicted on the left panels of Fig. 12 with the histogram concentrated at 0, confirming the inadequacies of both models. Conversely, the SW *p*-values of the RQRs for the fitted NB and ZINB models varied between 0 and 1 with about 96% of the *p*-values being above 0.05, as depicted on the right panels of Fig. 12, demonstrating the adequacies of both NB and ZINB models, although about 5% of the SW *p*-values are below 0.05.

To further illustrate RQR can be well approximately by a standard normal distribution in comparison with other types of residuals in the real data application, we calculated the mean, variance, skewness, kurtosis and the *p*-value of the SW normality test for the Pearson residual, deviance residual, MQR and RQR in the real data analysis. Note that for the SW normality test of the RQRs, the average of SW *p*-values based on 1000 replicated RQRs was presented to account for the fluctuation in the residuals introduced by randomization. As shown in Table 6, the results indicate that the means of the Pearson residuals, MQRs and RQRs are close to zero, but the means of the deviance residuals are consistently lower than zero under all models. The variances of Pearson residuals under all models are above one, and the variances of the deviance residuals and MQRs are all below one. By contrast, the variances of RQR are equal to one for all models, since the RQRs in the present study are all standardized. In terms of skewness and kurtosis, the Pearson residual, deviance residual, and MQR are right-skewed and heavy-tailed relative to a normal distribution, but RQRs are approximately symmetric with the tail shape close to a normal distribution under the NB and ZINB models. For testing the normality of the residuals based on the SW normality test, the results clearly showed that all models were rejected under the Pearson residual, deviance residual, and MQR. By contrast, RQRs confirm the adequacy of NB and ZINB models, with an average of SW *p*-values close to 0.5.

**Table 6:** Mean, variance, skewness, kurtosis and the *p*-value of the SW normality test for the Pearson residual, deviance residual, MQR and RQR in the real data analysis

	Model	Pearson	Deviance	MQR	RQR
Mean	Poisson	0.000	-0.292	0.043	-0.016
	NB	0.000	-0.311	0.066	-0.022
	ZIP	0.002	-0.004	0.236	0.153
	ZINB	0.000	-0.311	0.066	0.001
Variance	Poisson	1.495	0.789	0.555	1.000
	NB	1.040	0.466	0.425	1.000
	ZIP	1.145	0.927	0.657	1.000
	ZINB	1.040	0.466	0.425	1.000
Skewness	Poisson	3.507	2.179	2.467	0.508
	NB	3.524	1.980	2.058	0.029
	ZIP	3.546	2.009	2.290	0.361
	ZINB	3.524	1.980	2.058	0.019
Kurtosis	Poisson	18.225	5.189	7.500	1.259
	NB	17.649	3.273	3.504	-0.025
	ZIP	18.306	3.412	5.417	0.512
	ZINB	17.649	3.272	3.504	0.033
SW test	Poisson	0.000	0.000	0.000	0.000
	NB	0.000	0.000	0.000	0.452
	ZIP	0.000	0.000	0.000	0.000
	ZINB	0.000	0.000	0.000	0.459

Note that for the SW normality test of the RQRs, the average of SW *p*-values based on 1000 replicated RQRs was presented

## Discussion

Diagnosing regression models is crucial to ensure the validity of the results and implications that heavily rely on the tenability of the model assumptions. Pearson and deviance residuals and their corresponding *χ*^2^ tests are commonly used in practice. However, the use of these tools in count regression models are often not valid, since Pearson and deviance residuals do not have an asymptotic normal distribution under the correctly specified model. This paper reminisces that RQRs as a diagnostic tool to show that it has great advantages over other types of considered residuals for diagnosing count regression models, including zero-inflated count regression models.

Although using the QQ-plots for the Pearson residual, the deviance residual or MQR with simulated envelope could be used to check the model fit, visual inspection of the points falling outside of the simulated envelope can be subjective, and no single numerical measure of the overall model fit could be easily summarized based on such plots. A key strength of RQRs is that the plots of RQRs against covariates or fitted values contain important information concerning the inadequacy of different aspects of the model, which could not be easily reflected in the QQ plots with simulated envelopes. Further, the distribution of RQRs under the correctly specified count model is well approximated by a standard normal distribution. As a result, a numerical measure of overall model fit, i.e., SW normality test, can be readily derived, which was shown in our study to perform well for testing the overall model lack of fit.

Note that in our investigations, RQRs are standardized to have unit variance, as suggested by Klar and Meintanis (2012) [11]. Therefore, testing the normality of RQRs is essentially the same as testing standard normality in our investigation. Our study showed that Pearson, deviance, and MQR residuals for count regressions are not well approximated by a normal distribution. As such, testing standard normality of those residuals would be more stringent than testing normality, which might give even stronger evidence of the inadequacy of those types of residuals for diagnosing count regression models.

RQRs depend on a random uniformly distributed number *u*_*i*_ to convert the discrete cumulative distribution function into continuous values. Although the randomness in *u*_*i*_ may produce special patterns in the RQRs, we note that the chance that the pure random numbers will make any observable pattern decreases as the sample size *n* increases. Additionally, the influence from the randomness in *u*_*i*_ decreases as the number of possible values of *y*_*i*_ increases due to the shrinkage in the continuity gaps in the PMF. Nevertheless, as suggested by [10], multiple realizations of the RQRs are needed to ensure that any pattern shown in the RQRs is not caused by the randomness in *u*_*i*_. Although this offers a solution to alleviate the impact of the randomness in the RQRs, it is still desired to have a “non-random” overall GOF test *p*-value for the RQRs. Using the mean of the normality test *p*-values from multiple sets of the RQRs is a natural choice. Further research is needed to investigate the null distribution of the mean or other summary for replicated normality test *p*-values under the true model.

Further, although deviance residuals may not be appropriate for diagnosing counts regressions, they may be constructed for each systematic component (for example, location and dispersion) to assess specific goodness-of-fit. For example, Paula (2013) [35] used a normal probability plot with a simulated envelope for the deviance component residual for the mean and precision models in double generalized linear models [36]. Extension of randomized quantile residuals for each systematic component could be developed to give local information for assessing the specific goodness of fit. In addition, in generalized additive models for location, scale and shape proposed by Rigby and Stasinopoulos (2005) [37], the worm plot (a de-trended normal QQ-plot of the normalized quantile residuals) [38], a diagnostic tool for checking the residuals within different ranges of the explanatory variable(s), was proposed to identify regions (intervals) of the explanatory variable within which the model does not adequately fit the data. Such a plot could be constructed based on randomized quantile residuals for checking model inadequacies for count regression models.

In many applications, counts data are often clustered (i.e., longitudinal, spatial, or multilevel data) due to unmeasured cluster-level confounders. For modeling the complex dependence structure in these types of data, mixed-effects models are widely used (e.g., [39]). Programs to extend normal and non-normal regression models to clustered or longitudinal data are widely available (e.g., lme4 and mgcv packages in the R software, and glimmix and nlmixed procedures in the SAS software); however, model diagnosis for the mixed-effects models for counts data are still underdeveloped. Further development of extending the RQR method to examine the GOF of the mixed-effects models for counts data is underway by our research group, where the aforementioned data features are often encountered.

RQRs can also be extended as an alternative of the widely used posterior predictive diagnostics [40] for validating hierarchical Bayesian models in the Bayesian framework. However, extending RQRs to diagnose these complex models is non-trivial due to the optimistic bias of the posterior predictive diagnostics, where the actual observations are used twice for sequentially estimating parameters and testing the predictive distribution. The optimal bias could lead to posterior predictive *p*-values concentrating around 0.5 rather than being truly uniformly distributed (even after the randomness is applied). Leave-one-out cross-validation (LOOCV) is an alternative to the posterior predictive diagnostics. However, the actual LOOCV approach is time-consuming because several Markov chains are required in order to sample from each posterior distribution in which an observation is excluded as a test case. There have been numerous computational methods proposed to apply model diagnostics with the Markov chain samples from the posterior distribution based on the full dataset without the actual LOOCV being implemented [22–25, 41, 42]. It is possible to apply these methods to compute LOOCV RQRs in the Bayesian framework.

## Conclusion

In this paper, we demonstrated that the RQRs is approximately standard normally distributed under the correctly specified model, and their overall goodness-of-fit (GOF) test (i.e., Shapiro-Wilk (SW) normality test) is well-calibrated for diagnosing count regression models including zero-inflated models. Our simulation results indicate that the RQRs perform reasonably well to detect many forms of model misspecification: Non-linearity, zero-inflation, and over-dispersion. As expected, the statistical power of the RQRs for detecting model misspecification tends to be low for small sample sizes with minor deviations from the fitted models to the true models. Nevertheless, RQRs have substantive appeal in diagnosing counts regression models with moderate or large sample sizes and deviations between the fitted and true models.

## Appendix A: Supplementary materials

**Table 7:** Generalized variance inflation factor (GVIF) values for all the covariates

	GVIF	df	GVIF ^1/(2·*df*)^
Age	1.20	1.00	1.10
Race	1.19	1.00	1.09
Sex	1.21	1.00	1.10
Marital status	1.37	1.00	1.17
Education	1.28	1.00	1.13
Region	1.10	3.00	1.02
Family income	1.17	1.00	1.08
Employment status	1.09	1.00	1.05
Supplementary private insurance	1.45	1.00	1.20
Public insurance	1.40	1.00	1.18
health_excellent	1.80	1.00	1.34
health_average	1.84	1.00	1.36
Number of chronic conditions	1.18	1.00	1.09
Disability status	1.32	1.00	1.15

## Data Availability

The dataset analysed during the current study is available in the Journal of Statistical Software repository www.jstatsoft.org/article/view/v027i08, named as DebTrivedi.rda.zip.

## Abbreviations

RQR: Randomized quantile residuals
NB: Negative binomial
GOF: Goodness-of-fit
GLMs: Generalized linear models
ZIP: Zero-inflated Poisson
ZINB: Zero-inflated negative binomial
PMF: Probability mass function
CDF: Cumulative distributions function
MQR: Middle-point quantile residuals
QQ plot: Normal quantile-quantile plot
SW: Shapiro-Wilk
ED: Emergency department
LOOCV: Leave-one-out cross-validation VIF: Variance inflation factor
